# BeiDou Satellite Positioning Method Based on IoT and Edge Computing

**DOI:** 10.3390/s20030889

**Published:** 2020-02-07

**Authors:** Lina Wang, Rui Qiu

**Affiliations:** 1 School of Computer and Communication Engineering, University of Science and Technology Beijing, Beijing 100083, China; s20170652@xs.ustb.edu.cn; 2 Shunde Graduate School, University of Science and Technology Beijing, Foshan 528300, China

**Keywords:** Beidou navigation satellite, edge computing, load balancing, internet of things, pseudo-range difference

## Abstract

The BeiDou navigation satellite system (BDS) developed by China can provide users with high precision, as well as all-weather and real-time positioning and navigation. It can be widely used in many applications. However, new challenges emerge with the development of 5G communication system and Internet of Things (IoT) technologies. The BDS needs to be suitable for the large-scaled terminal scenario and provides higher positioning precision. In this paper, a BeiDou differential positioning method based on IoT and edge computing is proposed. The computational pressure on the data center is offloaded to the edge nodes when the massive positioning requests of IoT terminals need to be processed. To ensure the load balancing of the edge nodes, the resource allocation of the terminal positioning requests is performed with the improved genetic algorithm, thereby reducing the service delay of the entire edge network. Moreover, the optimized unscented Kalman filter based on the edge node (EUKF) algorithm is used to improve the positioning precision of IoT terminals. The results demonstrate that the proposed positioning method has better positioning performance which can provide the real-time positioning service for the large-scale IoT terminals.

## 1. Introduction

The BeiDou navigation satellite system (BDS) is a self-developed global navigation satellite system in China [1,2,3]. It has been widely used in military, civil navigation, intelligent monitoring and transportations, and marine fields, etc. [4,5]. However, challenges still remain in the BDS with the development of the 5G networking technologies and application requirements. The 5G communication system supports efficient access and management of large-scale Internet of Things (IoT) terminals [6]. Consequently, the BDS needs the rapid response time and high positioning precision to promote its development and competitiveness.

Differential positioning is the most widely used positioning algorithm and relies on centralized approaches. The reference station transmits the received satellite observations to the data center. Then, the data center processes the received data and computes correction information by eliminating the errors of the satellite orbit, clock, and ionosphere. The correction information can be transmitted to the terminals in real-time through satellites, digital broadcasting, and mobile communication system [7]. However, this method is easy to cause traffic overload and signal congestion in high-throughput and large-scale IoT terminals scenario. The data center cannot effectively process terminal requests in real-time.

Recently, there are a lot of researches to solve the problems of computational pressure and bandwidth constraints in the data center [8,9,10,11]. The authors of [8] proposed a method for broadcasting real-time kinematic (RTK) positioning data to mobile terminals through mobile communication networks, which can expand the scope of positioning service by the data center. Compared with cloud computing based on the data center, edge computing could undoubtedly reduce latency, which is very important for real-time service [9]. The authors of [10] investigated the task offloading problems of the ultra-dense network. To minimize the task duration, a scheme to offload the task on edge cloud or process locally was proposed. However, this method does not consider the terminals with more complex mobility. The authors of [11] designed an application-aware workload allocation scheme for edge computing-based IoT to minimize the response time of IoT application. It chooses cloudlet as the computing node. Since processing several types of IoT applications requires stronger computing ability, whereas the edge nodes do not need more.

Edge computing adds processing ability of task computing and data analyses to the network edge devices. Here, the main functionality of the edge nodes is data offloading, processing, and computing. Edge computing mainly has these benefits: It can reduce the computing load of the data center, it reduces the pressure of network bandwidth, improves the data processing efficiency of the IoT terminals, and it provides users with high reliability and low latency positioning service [12].

This paper proposes a BeiDou satellite positioning method based on IoT and edge computing, which can provide real-time, low latency, and high precision positioning service for massive IoT terminals. In this method, the upgraded cellular base stations are used as the edge nodes to offload the computational pressure on the data center. Additionally, the differential correction information is calculated and broadcast in real-time. To cope with the multi-access IoT terminals, an improved genetic task allocation algorithm is formulated, which minimizes the service delay while ensuring the load balancing of edge nodes. Then, the optimized unscented Kalman filter based on the edge node (EUKF) algorithm is used to improve the positioning precision of IoT terminals.

The remainder of this article is organized as follows. Section 2 introduces the pseudo-range differential positioning algorithms. Section 3 presents the proposed positioning method based on IoT and edge computing. The improved GA is used to address the load balancing issue and reduce the positioning delay. In addition, the optimized EUKF algorithm is used to improve the positioning precision. Section 4 provides the simulation results and analyses. Finally, the conclusions are drawn in Section 5.

## 2. Algorithm Principle

According to the difference of correction information from the reference stations, differential positioning can be divided into position differential positioning, pseudo-range differential positioning, and carrier phase differential positioning [13].

**Figure 1:**
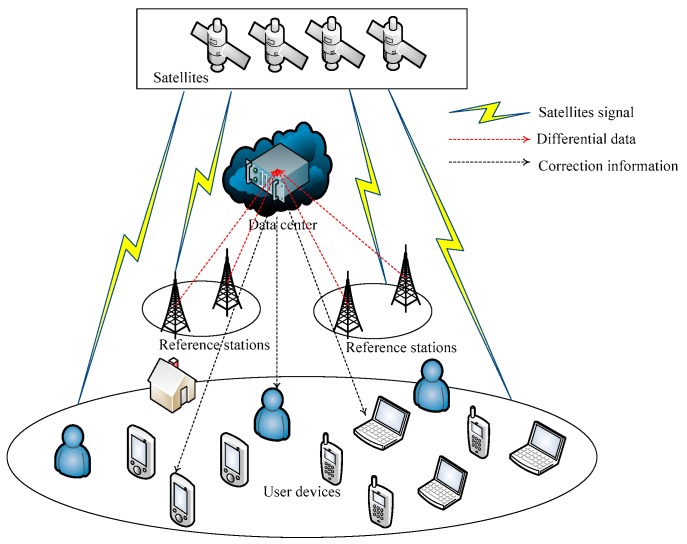
Differential positioning based on the data center.

Pseudo-range differential positioning is most widely used and can meet the positioning and navigation requirements of general IoT terminals. In short, the reference station receiver r measures the pseudo-ranges $P_{r}$ of all visible satellites, and calculates the corrections information according to the known position coordinate. Then, it broadcasts the corrections information of all satellites to the data center. The IoT terminals select an optimal set of four satellites to measure pseudo-ranges and the approximate coordinates of users, send the approximate coordinates to the data center, and receive the pseudo-range corrections from the nearest reference station. Finally, the corrected position coordinate is obtained according to the pseudo-range corrections [14,15]. The differential positioning based on the data center is shown in Figure 1.

The satellite position coordinate $\left(x^{s},\;y^{s},\;z^{s}\right)$ can be calculated by the received ephemeris data. The precise coordinate of the reference station $\left(x_{r},\;\;y_{r},\;\;z_{r}\right)$ can be obtained by the static positioning of the receiver after a long time. The geometric distance from reference station r to satellite s is given in Equation (1).

(1) $$R_{r}^{s}=\sqrt{{\left(x_{r}-x^{s}\right)}^{2}+{\left(y_{r}-y^{s}\right)}^{2}+{\left(z_{r}-z^{s}\right)}^{2}},$$

The pseudo-range is the measurement distance between the satellite and the receiver. Due to the influences of satellite ephemeris, clock, ionosphere, and troposphere, there is a measurement error. The observation equation of pseudo-range is given in Equation (2).

(2) $$\rho_{r}^{s}=R_{r}^{s}+c\delta t_{r}-c\delta t^{s}+\delta \rho_{r}+I_{r}+T_{r}+\varepsilon_{r},$$

where $\rho_{r}^{s}$ represents the pseudo-range from the reference station receiver r to the satellite s, $\delta t_{r}$ and $\delta t^{s}$ are the receiver clock error and the satellite clock error, $I_{r}$ and $T_{r}$ are the ionosphere error and the troposphere error, $\delta \rho_{r}$ is the satellite ephemeris error, *ε_r_* is the pseudo-range measurement noise, c is the speed of light. The parameters in Equation (2) all represent the length quantity.

The pseudo-range correction of the reference station $\Delta \rho_{r}^{s}$ is the difference between the pseudo-range measurement value and the geometric distance, given in Equation (3).

(3) $$\rho_{u}^{s}=R_{u}^{s}+c\delta t_{u}-c\delta t^{s}+\delta \rho_{u}+I_{u}+T_{u}+\varepsilon_{u},$$

The pseudo-range observation equation of the IoT terminal receiver u is given in Equation (4).

(4) $$\rho_{u}^{s}=R_{u}^{s}+c\delta t_{u}-c\delta t^{s}+\delta \rho_{u}+I_{u}+T_{u}+\varepsilon_{u},$$

Combined with the pseudo-range correction of reference station, the corrected pseudo-range observation equation is expressed as Equation (5).

(5) $$\begin{array}{ll} \rho_{cu}^{s} & =\rho_{u}^{s}-\Delta \rho_{r}^{s}\\ & =R_{u}^{s}+c\left(\delta t_{u}-\delta t_{r}\right)+\left(\delta \rho_{u}-\delta \rho_{r}\right)+\left(I_{u}-I_{r}\right)+\left(T_{u}-T_{r}\right)+\left(\varepsilon_{u}-\varepsilon_{r}\right) \end{array},$$

Under the condition of a short baseline, the ephemeris error between the two receivers is almost constant, namely $\delta \rho_{u}\approx \delta \rho_{r}$. Other correlated errors, such as ionospheric and tropospheric errors, are in the first order range, namely $I_{u}\approx I_{r}$, $T_{u}\approx T_{r}$. The measurement noise can be neglected, $\varepsilon_{u}\approx \varepsilon_{r}$. Then, the corrected pseudo-range observation equation can be expressed as Equation (6).

(6) $$\rho_{cu}^{s}=R_{u}^{s}+c\delta t_{u}=\sqrt{{\left(x_{u}-x^{s}\right)}^{2}+{\left(y_{u}-y^{s}\right)}^{2}+{\left(z_{u}-z^{s}\right)}^{2}}+c\delta t_{u},$$

There are four unknown numbers in Equation (6), that is the IoT terminal position coordinate $\left(x_{u},\;\;y_{u},\;\;z_{u}\right)$ and the receiver clock error $\delta t_{u}$. The precise coordinate of the IoT terminal can be calculated by the least square method, as long as four or more satellites are observed at the same time. 

However, this method centralizes the positioning request processing of IoT terminals to the data center, thereby resulting in the traffic overload. Therefore, edge computing is introduced into the positioning method. The edge nodes are used to offload the computing pressure on the data center, thereby providing effective and real-time positioning service.

## 3. Proposed Positioning Method Based on IoT and Edge Computing

### 3.1. Positioning System Architecture

The development of 5G communication technologies lays a foundation for the integration of navigation and communication. At the architecture level, navigation and communication equipment are integrated into one device. The base station in the cellular network is upgraded to serve as a reference station to observe navigation satellite signals. Combined with the existing and under construction continuous operation reference stations, the edge computing network is formed to provide navigation and positioning service for the IoT terminals. According to incomplete statistics, as of the beginning of 2017, China has established more than 6000 continuous operation reference stations.

Edge computing is a technology which conducts calculation at the network edge through a small data center closer to the terminals. The edge is the immediate first hop from the IoT device but not the IoT node itself, such as IoT gateways and base stations [16,17,18,19]. As an important part of the IoT, the base stations play a bridge role. It connects the IoT terminals and the cloud service. The continuous operation reference station situated at the IoT terminal side serves as an edge node which can provide high-precision positioning service with low latency, real-time interaction, mobility support, security, privacy for numerous deployed, and geographically dispersed IoT nodes.

**Figure 2:**
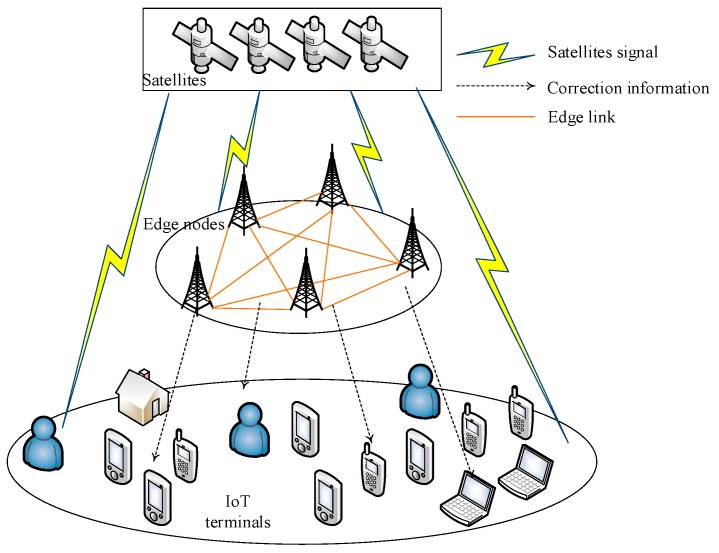
Positioning system architecture based on IoT and edge computing.

The positioning system architecture based on IoT and edge computing has three layers, that is the cloud layer, edge layer, and things layer, shown in Figure 2. The cloud layer includes a cloud server and data center. The edge layer includes a continuous operation reference station and base station network. The things layer includes IoT terminals and sensors. The edge layer and things layer participate in the positioning, whereas the cloud layer does not participate in the positioning, but only does the data collection, analysis, and processing work.

IoT node: The IoT node consists of sensors, devices, and terminals. The terminal initiates the positioning request. The GPS sensor measures the satellite signal and sends the measurement data to the edge node. The edge node calculates according to certain rules and policies. Finally, the terminal user obtains the positioning result.

Edge node: Edge node, which is equivalent to the small data center, can provide computing and storage resources to meet the positioning service requirements of various IoT terminals. The edge node can perform data preprocessing, simple data analysis and prediction, and send aggregated results to the cloud servers or IoT terminals. The edge nodes can communicate with each other, connect into the edge computing network, and carry out distributed computing. According to the approximate coordinate of the positioning terminal, the edge node dynamically generates the differential correction information to provide users with real-time and high-precision positioning results.

Cloud: The cloud uses the collected data by the IoT terminals. It can provide core service for the IoT terminals that include historical data analyses, data storage, and user behavior prediction. The location-based service can provide users with a more intelligent service based on the analysis results. The cloud service includes IoT terminal users tracking, configuration, analysis, reporting, authentication, and authorization service [20].

The BDS positioning method based on IoT and edge computing aims to solve the high time delay problem of centralized computing in the data center. The base station is used as the edge node to calculate the differential correction information. When the terminal initiates the positioning request, the nearest edge node receives the positioning request and calculates the differential correction information, and sends the final result to the terminal for positioning calculation.

### 3.2. Positioning Method

The IoT terminals send positioning requests and each terminal will choose the nearest edge node for positioning calculation. This scheme does not consider the load balancing problem of edge nodes, which will lead to overload and delay the increase of some edge nodes. Additionally, it is difficult to respond to the sudden changes in terminal requests. In view of numerous IoT terminals and the uneven geographical distribution, it is easy to obtain the optimal load balancing results and minimize the service delay by formulating the link scheduling problems. The final differential correction information result is sent to the IoT terminal by the nearest edge node through the Internet, and the accurate positioning result of the terminal can be obtained in a short time.

The edge computing network can be represented by a weighted directional graph G=(V,E), where $V=\left\{v_{1},\ldots ,v_{i},\ldots ,v_{m}\right\}$ is the set of edge nodes, and $E=\left\{e_{1,2},\ldots ,e_{i,j},\ldots ,e_{m-1,m}\right\}$ is the set of edge links. The edge link between $v_{i}$ and $v_{i}$ is $e_{i,j}$, and the communication delay of $e_{i,j}$ is $\tau_{i,j}$. Each edge node is granted with computing ability $r_{i}$. The set of positioning request tasks is $U=\left\{u_{1},\ldots ,u_{k},\ldots ,u_{n}\right\}$. The service delay processed by the whole computing task U in the edge computing network can be expressed as Equation (7).

(7) $$d=max\left[\frac{C_{i}}{r_{i}}+t_{k,i}+\tau_{i,j}x_{i,j}\right],$$

where $C_{i}$ is the request capacity on the edge node $v_{i}$, $\frac{C_{i}}{r_{i}}$ is the computation time on the edge node $v_{i}$. It can also indicate the waiting time of the task $u_{k}$. $t_{k,i}$ is the transmission delay from the IoT terminal k to the nearest edge node $v_{i}$. If the task $u_{k}$ is transmitted from edge node $v_{i}$ to $v_{j}$ and calculated at the edge node $v_{j}$, let $x_{i,j}=1$. Otherwise $x_{i,j}=0$, the task $u_{k}$ is calculated at the nearest edge node $v_{i}$.

Considering the mobility of the IoT terminal and the limited-service scope of edge nodes, the computing result backhaul delay can be expressed as Equation (8).

(8) $$d_{b}=\{\begin{array}{c} t_{k,i}^{\prime },\;t_{k,i}^{\prime }\leq \varepsilon \\ t_{k,i}^{\prime }+\tau_{i,j},\;t_{k,i}^{\prime }>\varepsilon \end{array},$$

where ε is the service range of the edge node $v_{i}$. If the IoT terminal k is within the coverage of edge computing node $v_{i}$, the differential correction information is transmitted from the edge computing node $v_{i}$ to the IoT terminal k. Otherwise, it is transmitted by the nearest edge node $v_{j}$ to the IoT terminal k.

The service delay of positioning request in the edge computing network is equal to the maximum delay of all IoT terminals. To achieve the goal of minimum delay of positioning request, it is necessary to minimize the objective function d. Hence, the objective function of the task allocation problem can be formulated as follows.

(9) $$\underset{i\in V,k\in U}{min}\left\{max\left[\frac{C_{i}}{r_{i}}+t_{k,i}+\tau_{i,j}x_{i,j}+d_{b}\right]\right\},$$

When positioning tasks are assigned among the edge nodes, each positioning request should be allocated to the nearest edge node to minimize the service delay. Positioning task assignment is initialized by assigning all positioning requests to the nearest edge node.

Due to the uneven distribution of terminals’ geographical location and the different computing ability of each edge node, the initialization of positioning requests may cause overload of some edge nodes. 

Reallocating the positioning requests on edge nodes to ensure load balancing can reduce the service delay of the whole system and maximize the throughput of the system. After the terminal positioning request is initialized, the average calculation time of the edge nodes is selected as the threshold. Then, the positioning requests exceeding the threshold φ are reallocated. On each edge node, there is the capacity $\Delta C_{i}$ used for the reallocated positioning requests. The task allocation problem can be represented as Equation (10).

(10) $$\underset{i\in V,k\in U}{min}\left\{max\left[\varphi +\frac{\Delta C_{i}}{r_{i}}+\tau_{i,j}x_{i,j}+d_{b}\right]\right\},$$

Currently widely used task allocation algorithms include genetic algorithm (GA) and ant colony algorithm (ACA). The ACA is more complex and requires a longer running time, so it is not suitable for real-time positioning in this paper. The GA [21] based on natural selection can solve large-scale combinatorial optimization problems and perform a parallel search. It has been proved to have robust search capabilities and could jump out local search space to achieve optimal solutions in global space. The best solution or secondary solutions are achieved by repeating employing three genetic operations, selection, crossover, and mutation. The improved GA proposed by this paper is used to redistribute positioning requests beyond the threshold φ to ensure the minimum service delay of the system. In order to reduce the execution time of the GA, the first generation of the chromosome is initialized as a solution space on the edge node whose calculation time is lower than the threshold φ. The hybrid selection method is used as a chromosome selection strategy. First, the top 20% best individuals are selected into the next generation directly, and set cp=0.2. Then, the roulette algorithm is used as a selection operation for the remaining 80% individuals. The roulette algorithm is as follows.

(1) Calculate the fitness value of each individual $f_{k}$.

(2) Calculate the probability of the selected individual k.

(11) $$P_{k}=\frac{f_{k}}{\sum_{k=1}^{n}f_{k}},$$

(3) Calculate the accumulation probability of an individual k.

(12) $$Q_{k}=\sum_{i=1}^{k}P_{k},$$

(4) Determine whether an individual is selected. Generate the random number between [0,1]. If $r<Q_{1}$, select the individual 1. If $Q_{k-1}<r\leq Q_{k}$, select the individual k.

The crossover operator is the most important step in the genetic algorithm. It can determine the global search ability of the algorithm. The individuals selected by the selection operation have high adaptability. Randomly select two of the above-mentioned individuals for crossover operation. Judge the generated random number rand and crossover probability $p_{c}=0.6$. If the crossover probability $p_{c}$ is large, the crossover operation is performed on two individuals to generate better individuals.

The mutation operation affects the diversity and local search ability of the population. In this paper, a single point mutation method is used to select one of the edge nodes randomly for gene mutation when the individual performs mutation operation. This randomness can enhance the local search ability of the algorithm, accelerate its convergence speed when the algorithm approaches the optimal solution, and reduce the immature convergence of the algorithm.

The above method reasonably assigns each positioning request to an edge node which calculates the differential correction information. It can effectively reduce positioning delay and provide real-time positioning services for terminals.

The load balancing degree of the edge computing network is measured by the standard deviation of task processing time (TSD) on each node [22]. For a good and stable load balancing performance, TSD should be low.

(13) $$\mathrm{TSD}=\sqrt{\frac{1}{n}\sum_{i=1}^{n}{\left(T_{i}-\bar{T}\right)}^{2}},\;\bar{T}=\frac{1}{n}\sum_{i=1}^{n}T_{i},$$

where i is the edge node, $T_{i}$ is the task processing time on the edge node i, $\bar{T}$ is the mean value of the time. Load balancing and service delay of the edge computing network affect the effectiveness of the task allocation algorithm.

The task allocation algorithm for the whole system is shown in Algorithm 1.
**Algorithm 1: The Task Allocation Algorithm**1.**Input:** List of terminals positioning requests and list of edge nodes2.**Output:** Minimum service delay3.**Initialize**$V,\;E,\;U,\;r_{i}$4.**Calculate**φ and $\Delta C_{i}$ for i∈V
5.**For** all i in edge nodes, i∈V
6. **Initialize**
$\Delta C_{i},\;cp,\;p_{c},\;CHROMOSOME,\;ITERATION$7. **Initialize** the first generation chromosome8. **While**
iteration<ITERATION
**do**9.  **Calculate** fitness $f_{k}$ of each chromosome in the previous generation10.  **Calculate** natural selection probability $P_{k}$
11.  **Copy** top 20% best individuals into next generation directly12.  The roulette algorithm is used to selection operation for the remaining individuals13.  **Crossover** operator14.  **Mutation** operator15.  **Calculate** the optimal allocation scheme and task processing time in this iteration16.  Generate the next generation chromosomes17. **End While**18.**End For**19.**Calculate** service delay of the whole system20.**Return** the optimal solution of service delay

After performing the task assignment algorithm, all terminals’ positioning requests are assigned to an edge node which is recorded as the master station. In the 5G scenario, there are lots of base stations used as edge nodes. The differential correction information of the approximate coordinates of the IoT terminal is calculated by using the master station in combination with at least two auxiliary stations around the IoT terminal [23]. Since the approximate coordinate of the IoT terminal $\left(x_{u}^{\prime },\;y_{u}^{\prime },\;z_{u}^{\prime }\right)$ is between several meters and ten meters apart from the real coordinate $\left(x_{u},\;y_{u},\;z_{u}\right)$, the differential correction information of the approximate coordinates can be used to correct the pseudo-range observation to obtain accurate IoT terminal positioning results. 

The 5G base station has a high density of stations, which is easily affected by multipath effects, resulting in a large noise of the observed signals. When the master station chooses the auxiliary station for calculation, auxiliary stations need to have good health, and the dilution of precision (DOP) is small and evenly distributed around the IoT terminal. DOP indicates the space distribution feature of the satellite when the receiver observes. The DOP value is smaller, the accuracy of navigation and positioning is higher. Otherwise, the navigation and positioning accuracy in the region becomes worse [24]. Position dilution of precision (PDOP), which is a measure of X, Y, Z position geometry, is generally less than three to obtain better positioning results [25]. If there are n available auxiliary stations in the edge computing network, the differential correction information $\Delta \rho_{mi}^{s}$ of auxiliary i relative to master station m is given in Equation (14).

(14) $$\Delta \rho_{mi}^{s}=\Delta R_{mi}+c\Delta \delta t_{mi}+\Delta \delta \rho_{mi}+\Delta I_{mi}+\Delta T_{mi}+\Delta \varepsilon_{mi},$$

For the approximation $u^{\prime }$ of the IoT terminal, the single difference between the master station m and $u^{\prime }$ can be expressed as Equation (15).

(15) $$\Delta \rho_{mi}^{s}=\Delta R_{mi}+c\Delta \delta t_{mi}+\Delta \rho_{mi}+\Delta I_{mi}+\Delta T_{mi}+\Delta \varepsilon_{mi},$$

Equation (15) can represent a linear function of the difference between the coordinates of the master station m and $u^{\prime }$.

(16) $$\Delta \rho_{mu^{\prime }}^{s}=\frac{\partial \Delta \rho }{\partial x}\left(x_{u}^{\prime }-x_{m}\right)+\frac{\partial \Delta \rho }{\partial y}\left(y_{u}^{\prime }-y_{m}\right)=a_{1}\left(x_{u}^{\prime }-x_{m}\right)+a_{2}\left(y_{u}^{\prime }-y_{m}\right),$$

Similarly, for auxiliary stations 1 and 2, we have

(17) $$\{\begin{array}{c} \Delta \rho_{m1}^{s}=a_{1}\left(x_{1}-x_{m}\right)+a_{2}\left(y_{1}-y_{m}\right)\\ \Delta \rho_{m2}^{s}=a_{1}\left(x_{2}-x_{m}\right)+a_{2}\left(y_{2}-y_{m}\right) \end{array},$$

The coordinates of the master station and the auxiliary station are known, and the pseudo-range single difference can be calculated by the observation data, and the solution coefficient is given in Equation (18).

(18) $$\left[\begin{array}{c} a_{1}\\ a_{2} \end{array}\right]={\left[\begin{array}{cc} x_{1}-x_{m} & y_{1}-y_{m}\\ x_{2}-x_{m} & y_{2}-y_{m} \end{array}\right]}^{-1}\left[\begin{array}{c} \Delta \rho_{m1}^{s}\\ \Delta \rho_{m2}^{s} \end{array}\right],$$

Using Equation (16), we have

(19) $$\begin{array}{ll} \Delta \rho_{mu^{\prime }}^{s} & =a_{1}\left(x_{u}^{\prime }-x_{m}\right)+a_{2}\left(y_{u}^{\prime }-y_{m}\right)\\ & =\left[\begin{array}{cc} x_{u}^{\prime }-x_{m} & y_{u}^{\prime }-y_{m} \end{array}\right]{\left[\begin{array}{cc} x_{1}-x_{m} & y_{1}-y_{m}\\ x_{2}-x_{m} & y_{2}-y_{m} \end{array}\right]}^{-1}\left[\begin{array}{c} \Delta \rho_{m1}^{s}\\ \Delta \rho_{m2}^{s} \end{array}\right] \end{array},$$

Then, the corrected pseudo-range observation equation of terminal u is represented as Equation (20).

(20) $$\begin{array}{ll} \rho_{cu}^{s} & =\rho_{u}^{s}-\Delta \rho_{mu^{\prime }}^{s}\\ & =R_{u}^{s}+c\left(\delta t_{u}-\delta t_{u}^{\prime }\right)+\left(\delta \rho_{u}-\delta \rho_{u}^{\prime }\right)+\left(I_{u}-I_{u}^{\prime }\right)+\left(T_{u}-T_{u}^{\prime }\right)+\left(\varepsilon_{u}-\varepsilon_{u}^{\prime }\right) \end{array},$$

Eliminate errors with a strong spatial correlation, such as ionosphere, troposphere, satellite orbit, and clock errors. The IoT terminal approximate coordinate is $\left(x_{u}^{\prime },\;y_{u}^{\prime },\;z_{u}^{\prime }\right)$, and the geometric distance to satellite is given in Equation (21).

(21) $$R_{u}^{\prime }=\sqrt{{\left(x_{u}^{\prime }-x^{s}\right)}^{2}+{\left(y_{u}^{\prime }-y^{s}\right)}^{2}+{\left(z_{u}^{\prime }-z^{s}\right)}^{2}},$$

According to the approximate coordinate of the IoT terminal, the distance difference can be expressed by the coordinate difference. Since the distance between the two coordinates is very close, the higher derivative of Taylor expansion is almost zero, and only the first derivative is retained. The error equation of the corrected pseudo-range observation equation is given in Equation (22).

(22) $$v_{uu^{\prime }}^{s}=e_{x}\Delta x+e_{y}\Delta y+e_{z}\Delta z+c\delta t_{u}+\left(R_{u^{\prime }}^{s}-\rho_{u}^{s}+\Delta \rho_{mu^{\prime }}^{s}\right),$$

The number of satellites is n, the corresponding error equations is given in Equation (23).

(23) V=AδX+L,

where

(24) $$A=\left[\begin{array}{cccc} e_{x}^{1} & e_{y}^{1} & e_{z}^{1} & 1\\ e_{x}^{2} & e_{y}^{2} & e_{z}^{2} & 1\\ \vdots & \vdots & \vdots & \vdots \\ e_{x}^{n} & e_{y}^{n} & e_{z}^{n} & 1 \end{array}\right],\;\delta X=\left[\begin{array}{c} \Delta x\\ \Delta y\\ \Delta z\\ c\delta t_{u} \end{array}\right],\;L=\left[\begin{array}{c} R_{u^{\prime }}^{1}-\rho_{u}^{1}+\Delta \rho_{mu^{\prime }}^{1}\\ R_{u^{\prime }}^{2}-\rho_{u}^{2}+\Delta \rho_{mu^{\prime }}^{2}\\ \vdots \\ R_{u^{\prime }}^{n}-\rho_{u}^{n}+\Delta \rho_{mu^{\prime }}^{n} \end{array}\right],$$

When four or more satellites are observed, the least square solution can be obtained by the weighted least square, where the mean value of L is 0 and the variance matrix is Q.

(25) $$\left[\begin{array}{c} \Delta x\\ \Delta y\\ \Delta z\\ c\delta t_{u} \end{array}\right]={\left(A^{T}Q^{-1}A\right)}^{-1}A^{T}Q^{-1}L,$$

The covariance matrix of the least squares estimator is $var\left(\delta X_{LS}\right)={\left(A^{T}Q^{-1}A\right)}^{-1}$. Finally, the accurate coordinate of the IoT terminal is obtained.

(26) $$\left[\begin{array}{c} x_{u}\\ y_{u}\\ z_{u} \end{array}\right]=\left[\begin{array}{c} x_{u}^{\prime }+\Delta x\\ y_{u}^{\prime }+\Delta y\\ z_{u}^{\prime }+\Delta z \end{array}\right],$$

**Figure 3:**
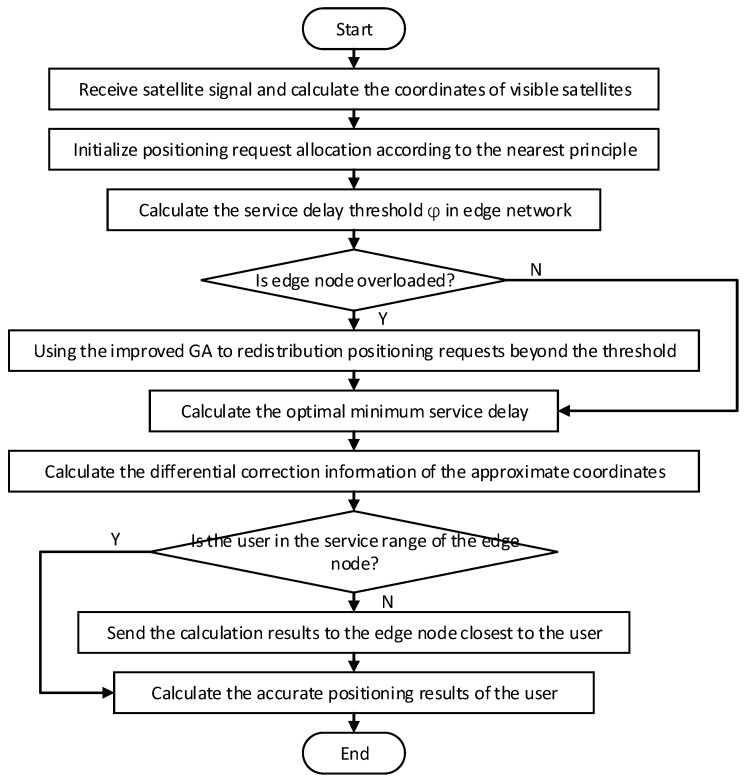
The flowchart of the BeiDou positioning method based on IoT and edge computing.

The flowchart of the BeiDou positioning method based on IoT and edge computing is shown in Figure 3.

### 3.3. Optimized Positioning Method

The least squares method described above is simple in calculation and the positioning results at each time are independent of each other, so the positioning error is large and unstable. Unscented Kalman filter (UKF) [26] is a recursive algorithm which is suitable for nonlinear systems and has the advantages of small calculation and high positioning accuracy. The UKF algorithm reduces the influence of errors on positioning accuracy through filters, enabling accurate target tracking.

Consider a nonlinear system as follows:

(27) $$\{\begin{array}{c} X_{k}=f\left(X_{k-1},W_{k-1}\right)\\ Z_{k}=h\left(X_{k-1},V_{k-1}\right) \end{array},$$

where f represents a nonlinear state function and h represents a nonlinear measurement function. $W_{k}$ and $V_{k}$ are mutually independent white noises with the mean of 0 and covariance matrices $Q_{k}$ and $R_{k}$, respectively.

In order to ensure the unbiasedness of the estimation, the initial value of the filtering is

(28) $$\begin{array}{c} \hat{X}=EX_{0}\\ P_{0}=E\left[\left(X_{0}-{\hat{X}}_{0}\right){\left(X_{0}-{\hat{X}}_{0}\right)}^{T}\right] \end{array},$$

The core of UKF is unscented transformation, which uses sigma points to approximate the Gaussian distribution of nonlinear systems, and the obtained mean and covariance have higher precision. The *n*-dimensional random variable X with mean $\bar{X}$ and covariance $P_{XX}$ can be approximated by sigma points.

(29) $$\{\begin{array}{c} \chi^{\left(0\right)}=\bar{X}\\ \chi^{\left(i\right)}=\bar{X}+{\left(\sqrt{\left(n+\lambda \right)P_{XX}}\right)}_{\left(i\right)},i=1,2,\ldots ,n\\ \chi^{\left(i\right)}=\bar{X}-{\left(\sqrt{\left(n+\lambda \right)P_{XX}}\right)}_{\left(i-n\right)},i=n+1,n+2,\ldots ,2n \end{array},$$

The corresponding weights of the sigma points are given in Equation (30).

(30) $$\{\begin{array}{c} W_{m}^{\left(0\right)}=\frac{\lambda }{n+\lambda }\\ W_{c}^{\left(0\right)}=\frac{\lambda }{n+\lambda }+1-\alpha^{2}+\beta \\ W_{m}^{\left(i\right)}=W_{c}^{\left(i\right)}=\frac{\lambda }{n+\lambda },i=1,2,\ldots ,2n \end{array},$$

where m is the mean and c is the covariance.

Calculate the sigma points at time k−1.

(31) $$\{\begin{array}{c} {\tilde{\chi }}_{k-1}^{\left(0\right)}={\hat{X}}_{k-1}\\ {\tilde{\chi }}_{k-1}^{\left(i\right)}={\hat{X}}_{k-1}+\sqrt{n+\lambda }{\left(\sqrt{P_{k-1}}\right)}_{\left(i\right)},i=1,2,\ldots ,n\\ {\tilde{\chi }}_{k-1}^{\left(i\right)}={\hat{X}}_{k-1}-\sqrt{n+\lambda }{\left(\sqrt{P_{k-1}}\right)}_{\left(i-n\right)},i=n+1,n+2,\ldots ,2n \end{array},$$

Calculate one-step prediction at time k.

(32) $$\begin{array}{c} \chi_{k/k-1}^{*\left(i\right)}=f\left[\chi_{k-1}^{\left(i\right)}\right],i=0,1,2,\ldots ,2n\\ {\hat{X}}_{k/k-1}=\sum_{i=0}^{2n}W_{m}^{\left(i\right)}\chi_{k/k-1}^{*\left(i\right)}\\ P_{k/k-1}=\sum_{i=0}^{2n}W_{c}^{\left(i\right)}\left[\chi_{k/k-1}^{*\left(i\right)}-{\hat{X}}_{k/k-1}\right]{\left[\chi_{k/k-1}^{*\left(i\right)}-{\hat{X}}_{k/k-1}\right]}^{T}+Q_{k-1} \end{array},$$

Calculate one-step prediction sigma points at time k.

(33) $$\begin{array}{c} \chi_{k/k-1}^{\left(0\right)}={\hat{X}}_{k/k-1}\\ \chi_{k/k-1}^{\left(i\right)}={\hat{X}}_{k/k-1}+\sqrt{n+\lambda }{\left(\sqrt{P_{k/k-1}}\right)}_{\left(i\right)},i=1,2,\ldots ,n\\ \chi_{k/k-1}^{\left(i\right)}={\hat{X}}_{k/k-1}-\sqrt{n+\lambda }{\left(\sqrt{P_{k/k-1}}\right)}_{\left(i-n\right)},i=n+1,n+2,\ldots ,2n\\ {\mathbb{Z}}_{k/k-1}^{\left(i\right)}=h\left[\chi_{k/k-1}^{\left(i\right)}\right],i=0,1,2,\ldots ,2n\\ {\hat{Z}}_{k/k-1}^{\left(i\right)}=\sum_{i=0}^{2n}W_{m}^{\left(i\right)}{\mathbb{Z}}_{k/k-1}^{\left(i\right)} \end{array},$$

Update the filter value.

(34) $$\begin{array}{c} P_{\left(XZ\right)k/k-1}=\sum_{i=0}^{2n}W_{c}^{\left(i\right)}\left[\chi_{k/k-1}^{\left(i\right)}-{\hat{X}}_{k/k-1}\right]{\left[{\mathbb{Z}}_{k/k-1}^{\left(i\right)}-{\hat{Z}}_{k/k-1}\right]}^{T}\\ P_{\left(ZZ\right)k/k-1}=\sum_{i=0}^{2n}W_{c}^{\left(i\right)}\left[{\mathbb{Z}}_{k/k-1}^{\left(i\right)}-{\hat{Z}}_{k/k-1}\right]{\left[{\mathbb{Z}}_{k/k-1}^{\left(i\right)}-{\hat{Z}}_{k/k-1}\right]}^{T}+R_{k}\\ K_{k}=P_{\left(XZ\right)k/k-1}P_{\left(ZZ\right)k/k-1}^{-1}\\ {\hat{X}}_{k}={\hat{X}}_{k/k-1}+K_{k}\left[Z_{k}-{\hat{Z}}_{k/k-1}\right]\\ P_{k}=P_{k/k-1}-K_{k}P_{\left(ZZ\right)k/k-1}K_{k}^{T} \end{array},$$

Combining the positioning model based on IoT and edge computing, an unscented Kalman filter based on the edge node (EUKF) is proposed in this paper. Considering the limited computing ability of the IoT terminal, the edge nodes are used to calculate the positioning results and sent to the IoT terminals. In order to improve the positioning accuracy, multiple edge nodes around the IoT terminals are used for coordinated positioning, and the positioning result is transmitted by the master station to the IoT terminal.

Independent filtering estimation of the IoT terminals is conducted by each edge node. The states of the N edge nodes are estimated as ${\hat{X}}_{1},{\hat{X}}_{2},\ldots ,{\hat{X}}_{N}$, and the corresponding estimated error covariances $P_{11},P_{22},\ldots ,P_{NN}$. The final filtered result can be expressed as Equation (35).

(35) $${\hat{X}}_{f}=A_{1}{\hat{X}}_{1}+A_{2}{\hat{X}}_{2}+\cdots +A_{N}{\hat{X}}_{N},$$

The combined measurement update can be expressed as

(36) $$\begin{array}{c} {\hat{X}}_{k}={\hat{X}}_{k/k-1}+\sum_{i}K_{ik}\left[Z_{ik}-{\hat{Z}}_{i,k/k-1}\right]\\ P_{k}=P_{k/k-1}-\sum_{i}K_{ik}P_{\left(ZZ\right)i,k/k-1}K_{ik}^{T} \end{array},$$

Each recursion of the EUKF uses the edge node around the master station to correct the measurement error. The error covariance matrix can be expressed as Equation (37).

(37) $$P_{\left(ZZ\right)i,k/k-1}=\sum_{i=0}^{2n}W_{c}^{\left(i\right)}\left[{\mathbb{Z}}_{k/k-1}^{\left(i\right)}-{\hat{Z}}_{k/k-1}\right]{\left[{\mathbb{Z}}_{k/k-1}^{\left(i\right)}-{\hat{Z}}_{k/k-1}\right]}^{T}+A_{i}R_{ik},$$

where $A_{i}$ is a weighted matrix, which can be determined by the distance from the edge node to the IoT terminal and the confidence of the edge node. The weighted matrix $A_{i}$ is given in Equation (38).

(38) $$A_{i}=diag\left(a_{1},a_{2},\ldots ,a_{n}\right),$$

The error covariance directly affects the gain of the filter, and the filter gain can be expressed as Equation (39).

(39) $$K_{ik}=P_{\left(XZ\right)i,k/k-1}P_{\left(ZZ\right)i,k/k-1}^{-1},$$

If the distance from the edge node to the IoT terminal is far and the confidence of the edge node is low, the value of $A_{i}$ is large, and the filter gain is correspondingly reduced, thereby reducing the influence of system error on the positioning accuracy.

The state vector of the IoT terminal is given in Equation (40).

(40) $$X={\left[x,y,z,\dot{x},\dot{y},\dot{z},\ddot{x},\ddot{y},\ddot{z},c\delta t_{u}\right]}^{T},$$

Equation (40) includes the terminal’s three-dimensional position coordinate, velocity, acceleration, and receiver clock difference. The equation of state for the IoT terminal is given in Equation (41).

(41) $$X_{k+1}=\Phi X_{k}+W_{k},$$

where

(42) $$\Phi =\left[\begin{array}{cccc} \Phi_{x} & O_{3} & O_{3} & O_{3}\\ O_{3} & \Phi_{y} & O_{3} & O_{3}\\ O_{3} & O_{3} & \Phi_{z} & O_{3}\\ O_{3} & O_{3} & O_{3} & 1 \end{array}\right],\;\Phi_{x}=\left[\begin{array}{ccc} 1 & T & T^{2}/2\\ 0 & 1 & T\\ 0 & 0 & 1 \end{array}\right],$$

where T indicates the adoption time. According to the above model, the position coordinates of the IoT terminal can be solved.

## 4. Results and Analysis

In this paper, OEM6^®^ Family Firmware is used to receive satellite data. NovAtel Connect provides a graphical interface for establishing communication, controlling, and monitoring the operation of the NovAtel receiver. The receiver software can track satellites indicator, dilution of precision, positioning results, etc. The satellite position coordinates and the positioning result of the IoT terminal can calculate by the ephemeris and observation data collected by the receiver.

**Figure 4:**
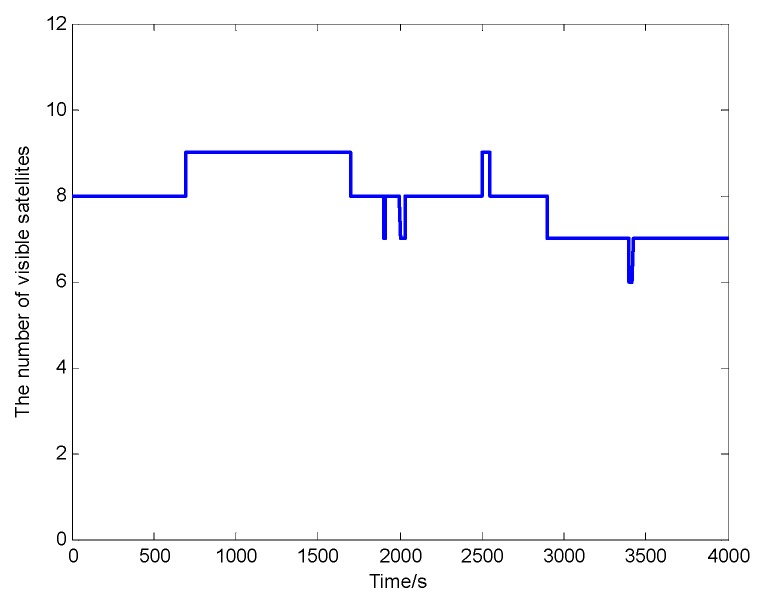
The number of visible satellites.

**Figure 5:**
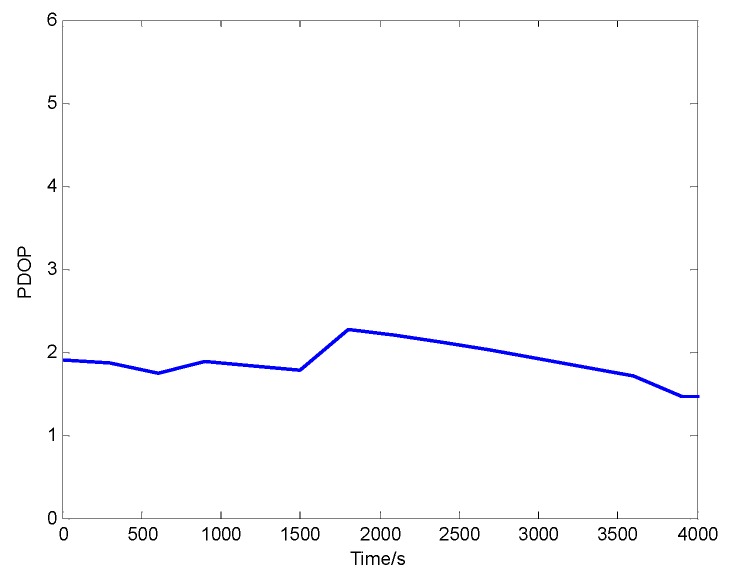
PDOP.

The observation of the visible satellites is shown in Figure 4. During the observation period, the number of the visible satellites meets the basic requirements of positioning calculation. The PDOP representing the geometric distribution of the satellites in the sky is shown in Figure 5. The PDOP value is less than three, therefore the accurate positioning results can be calculated using the observed satellite data.

This paper uses MATLAB for simulations. According to the average coverage of a 5G, the base station is 500 m, 20,000 base stations in Beijing are selected as edge nodes. Assume that the edge nodes have the same computing power, and the terminals’ positioning requests are generated randomly. The parameters of GA are set as follows: $cp=0.2,\;p_{c}=0.6$. The number of chromosomes is 100 and the number of iterations is 100. Use the real data collected by the NovAtel receiver to calculate the positioning results of the IoT terminals.

### 4.1. Positioning Accuracy

According to the observed satellite ephemeris data and pseudo-range information, the satellite position coordinates are calculated. The IoT terminals use the above-mentioned difference positioning algorithm based on IoT and edge computing to solve the terminals’ precise coordinates. 

**Figure 6:**
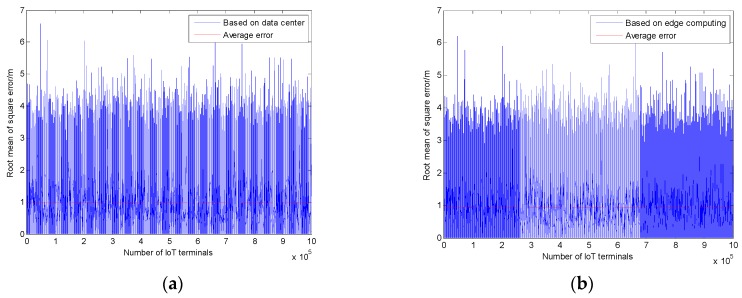
Average positioning errors of the positioning methods based on data center and edge computing respectively: (**a**) Average positioning errors based on the data center; (**b**) average positioning errors based on edge computing.

Figure 6 shows the root mean of square error of the differential positioning method based on the data center and edge computing, respectively. It can be seen from Figure 6a that the average positioning error based on the data center is 0.96268 m. Additionally, the average error based on edge computing is 0.95511 m, as shown in Figure 6b. The accuracy of the two methods is similar, but the average positioning error based on edge computing has 0.00757 m improvement. The result demonstrates that the proposed positioning method in this paper can provide the accurate positioning services for large-scale IoT terminals.

**Figure 7:**
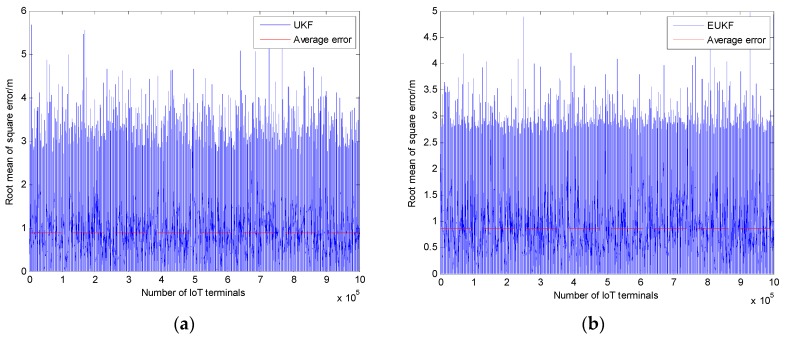
Positioning errors of the UKF and EUKF algorithms: (**a**) Positioning errors of the UKF algorithm; (**b**) positioning errors of the EUKF algorithm.

The comparisons of the positioning errors of the UKF and EUKF algorithms are shown in Figure 7, respectively. It can be seen from Figure 7a that the average positioning error of the UKF algorithm is 0.88389 m. Additionally, the average positioning error of the EUKF algorithm is 0.87495 m, as shown in Figure 7b. It can be known from Figure 6 and Figure 7 that the UKF and EUKF algorithms have higher and more stable positioning accuracy. However, the average positioning accuracy of the EUKF algorithm is 0.00894 m and it is higher than that of the UKF algorithm.

**Figure 8:**
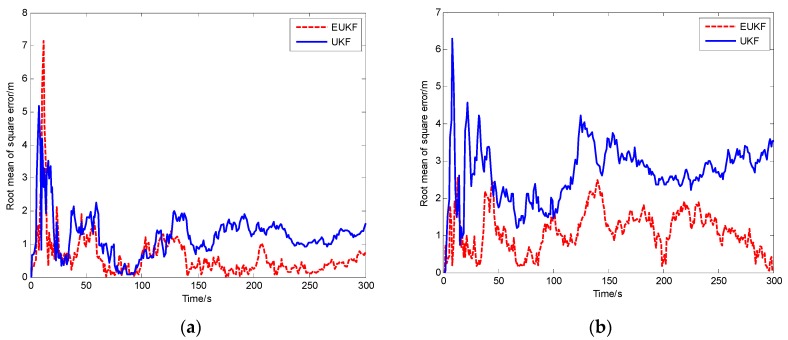
Errors of dynamic positioning: (**a**) Open environment; (**b**) ambiguous environment.

Then, the positioning errors under different environments are shown in Figure 8. The results show that the EUKF algorithm uses the edge nodes to eliminate the system error and its positioning accuracy is higher than that of the UKF algorithm. Comparing Figure 6b and Figure 7b, the average positioning error of the EUKF algorithm has 0.08016 m improvement than the positioning method based on edge computing.

### 4.2. Service Delay

**Figure 9:**
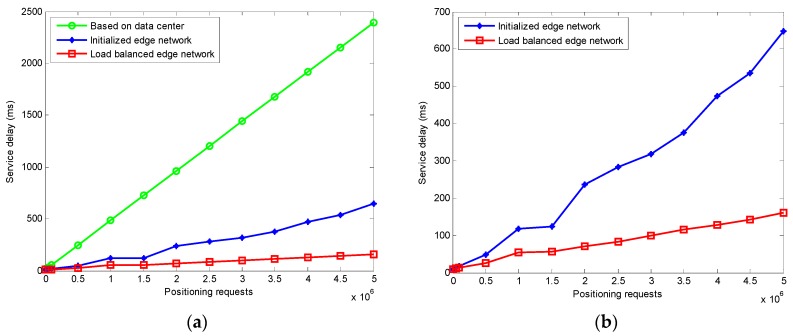
Service delay: (**a**) Comparisons of service delay among different methods; (**b**) service delay before and after load balancing.

The results of the service delay are shown in Figure 9. The service delay is the delay of the last IoT terminal to complete the positioning in the entire edge network. In Figure 9a, the positioning algorithm based on edge computing offloads the computational pressure of the data center. The differential correction information is solved by the edge node close to the IoT terminal, which mitigates the problem of the large calculation amount, bandwidth limitation and high throughput of the data center, and reduces the maximum service delay for the entire system. If the nearest edge node receives the positioning requests and calculates the differential correction information after initialization, the service delay will increase due to the imbalance of the IoT terminals’ positioning requests, as shown in Figure 9b. However, the edge computing network with improved GA can achieve the load balancing. The overloaded terminal positioning request on the edge node is reallocated, and the service delay is reduced.

**Figure 10:**
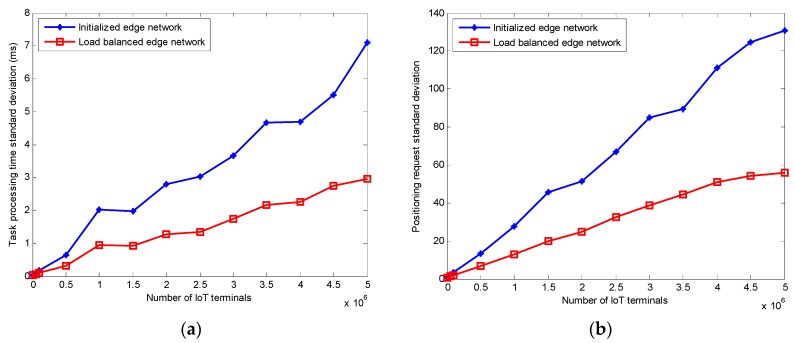
Load balancing performance: (**a**) Task processing time standard deviation; (**b**) positioning request standard deviation.

Figure 10 shows the load balancing performance of the edge network before and after executing the improved GA. It can be seen that with the increases of the number of IoT terminals, the improved positioning algorithm can balance the IoT terminals’ positioning requests and reduce the task processing time of the whole edge computing network.

**Figure 11:**
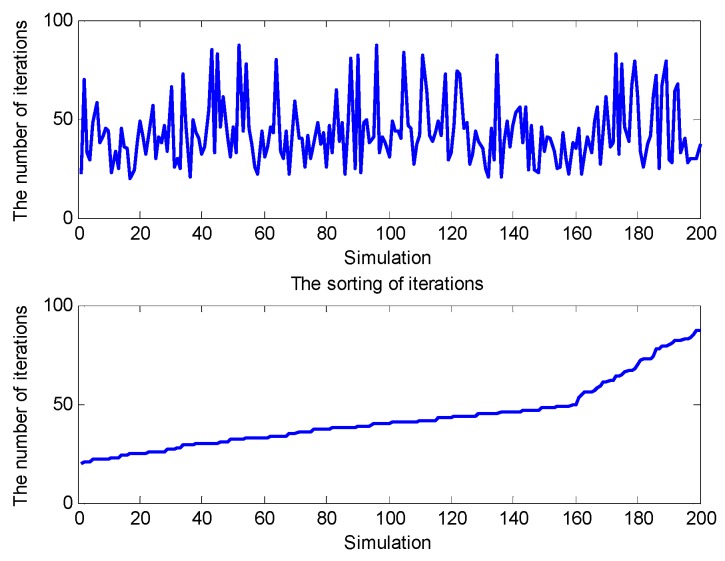
The number of iterations of 200 simulations.

Figure 11 shows the number of iterations of 200 simulations. The average number of iterations is 43.18. The results demonstrate that the proposed positioning algorithm has better search performance and can converge to the optimal result quickly.

## 5. Conclusions

With the increasing of the IoT terminals, traditional positioning methods based on the data center cannot provide high reliability and low latency positioning service because of high throughput and limited bandwidth. This paper proposes a BeiDou satellite positioning method based on IoT and edge computing, which initializes the positioning requests of the IoT terminals at the nearest edge node and reduces the system service delay on the basis of the improved GA to ensure the load balancing of the entire edge computing network. This method effectively reduces the calculation pressure on the data center and improves the calculation efficiency and real-time response speed of the system. The optimized EUKF algorithm can effectively reduce the influence of the system error on positioning accuracy by using edge nodes, which can improve the positioning accuracy of users and ensure the stability of positioning. The simulation results demonstrate that the proposed positioning method based on IoT and edge computing can provide real-time and accurate positioning services for the large-scale IoT terminals.
